# Single nucleotide polymorphisms of the *APC *gene and colorectal cancer risk: a case-control study in Taiwan

**DOI:** 10.1186/1471-2407-6-83

**Published:** 2006-03-29

**Authors:** Shee-Ping Chen, Shih-Tzu Tsai, Shu-Wen Jao, Yen-Lun Huang, Yu-Chen Chao, Yi-Lin Chen, Chang-Chieh Wu, Shinn-Zong Lin, Horng-Jyh Harn

**Affiliations:** 1 Institute of Medical Sciences, Buddhist Tzu Chi University: 703, Section 3, Chung-Yang Road, 970 Hualien, Taiwan; 2 Neuro-Medical Scientific Center Departments, Buddhist Tzu Chi General Hospital, Hualien, Taiwan; 3 Department of Family Medicine, Buddhist Tzu Chi General Hospital, Hualien, Taiwan; 4 Department of Surgery, Tri-Service General Hospital, Taipei, Taiwan; 5 Department of pathology, Buddhist Tzu Chi General Hospital, Hualien, Taiwan

## Abstract

**Background:**

Colorectal cancer (CRC), which has become especially prevalent in developed countries, is currently the third highest cause of cancer mortality in Taiwan. Mutation of the *adenomatous polyposis coli *(*APC*) gene, a tumour suppressor, is thought to be an early event in colorectal tumourigenesis. To date, however, no large-scale screening for *APC *gene variants in Chinese subjects has been performed. The present study was undertaken to identify *APC *gene variants that are significantly associated with the occurrence of CRC in Taiwanese subjects.

**Methods:**

In order to compare the genotype distribution of variant sites, the full-length *APC *genes of 74 healthy individuals and 80 CRC patients were sequenced.

**Results:**

Among the 154 Taiwanese subjects examined in this study, three new mutations, but no previously reported mutations, were found. One deletion at codon 460 leading to a frameshift and two missense mutations resulting in p.V1125A and p.S1126R substitutions were identified. Additionally, three high risk genotypes associated with three single nucleotide polymorphisms and one low risk genotype at codon 1822 were identified.

**Conclusion:**

The findings of this case-control study are consistent with the proposal that Taiwanese subjects differ from other subjects with respect to phenotypic presentation of *APC *and CRC risk.

## Background

Colorectal cancer (CRC) has increased in incidence over the past two to three decades and has become especially prevalent in developed countries. For example, CRC is currently the second most common form of cancer and the third leading cause of cancer mortality in the United States [1]. In Taiwan, CRC has become the fourth most common form of cancer and the third leading cause of death due to cancer. Approximately 10% of CRCs are attributable to genetic alterations whereas 90% occur sporadically [2].

Familial adenomatous polyposis (FAP), a disease inherited as an autosomal dominant, is characterized by the development of multiple colorectal polyps and possesses the potential to progress to CRC. FAP is now recognized to be caused by a functional mutation in the *adenomatous polyposis coli *(*APC*) gene [3,4]. The *APC *gene is located at 5q21-q22, contains 15 exons, has a corresponding mRNA of approximately 10 kb, and encodes a large protein with multiple functional domains. The gene product has been linked with several important processes including cell adhesion, maintenance of cytoskeletal integrity and chromosomal stability, and regulation of the cell cycle [5]. Previous findings are consistent with the proposals that the *APC *gene functions as a tumour suppressor and that somatic mutations in the *APC *gene predispose humans to development of FAP. The loss of APC function is currently thought to represent an event critical to colorectal tumourigenesis [6].

Although CRC attributable to mutations of the *APC *gene that lead to development of FAP represents less than 1% of all CRCs, *APC *germline mutations have close to 100% penetrance [7]. Exon 15 of the *APC *gene encompasses 75% of the gene coding sequence and is the most common mutation site. Over 98% of mutations in this region are either nonsense or frameshift mutations. The codons 1061 and 1309 are the most common germline mutation sites, and mutations at either site often result in a frameshift [8,9]. In addition, two missense germline mutations of *APC *are frequently reported. The first is the p.I1307K variant which is found in Ashkenazi Jews and is associated with a several-fold increased risk for development of multiple adenomas and CRC. The second is p.E1317Q, the mutation site of which lies between the first and second 20-amino acid β-catenin binding sites. This mutation has been proposed to suppress the APC/β-catenin pathway, thereby predisposing to the development of adenomas [10-12].

Somatic mutations in the *APC *gene are found in over 85% of sporadic colorectal adenomas and carcinomas [13]. More than 60% of all such mutations are found within the sequence between codons 1286 and 1513 and termed the mutation cluster region (MCR) [14]. Two hotspots for somatic mutations are found at codons 1286 and 1450; mutations at these sites often result in stop codons [8,9]. Mutations in regions of the MCR encoding the 20-amino acid β-catenin binding sites may result in functional defects in the APC protein [12].

To improve current understanding of the genetics and biological functions of APC, additional screening of the gene for single nucleotide polymorphisms (SNPs) or missense mutations is needed. Furthermore, recognition of such mutations is essential in determining the inherited risks for CRC. *APC *gene variants may present as population-specific. To date, however, no large-scale screening for *APC *gene variants in Chinese subjects has been conducted. The present case-control study was performed to characterize SNP mutations of the *APC *gene in the Taiwanese population and to identify the variants that are significantly associated with the occurrence of CRC.

## Methods

### Subjects samples

Health examinations were performed for 74 individuals at the Hualien Tzu Chi Medical Center and for 80 colorectal cancer patients at the Tri-Service General Hospital. All subjects were screened for colorectal tumors by colonscopy and none of them has the family history of CRC. Written informed consent was obtained from all subjects, and the study protocol was approved by The Protection of Human Subjects Institutional Review Board Tzu-Chi University and Hospital, IRB093-54.

The study group was comprised of 80 colorectal cancer patients and 74 healthy adults. The patient group consisted of 40 (50%) males of 63.6 ± 12.5 years of age (mean ± SD; range, 35–95 years) and 40 (50%) females of 59.2 ± 11.0 years of age (mean ± SD; range, 37–80 years). The mean age ± SD for all patients was 61.5 ± 12.0 years (range, 35–95 years). The health examination group consisted of 34 (45.3%) males of 55.8 ± 11.5 years of age (mean ± SD; range, 32–84 years) and 41 (54.7%) females of 53.0 ± 8.7 years of age (mean ± SD; range, 39–72 years). The mean age ± SD for all healthy adults was 54.3 ± 10.1 years (range, 32–84 years). In the patient group, there were 16 (20%) subjects with proximal tumors, 26 (32.5%) subjects with distal tumors, 38 (47.5%) subjects with rectal tumors, and there were 8 (10%) subjects with colorectal tumors in Duke's stage A, 24 (30%) in Duke's stage B, 38 (47.5%) in Duke's stage C and 10 (12.5%) in Duke's stage D.

### DNA extraction

Genomic DNA was extracted from 10 ml of whole blood through use of the Puregene Genomic DNA Purification Kit (Gentra Systems, Inc., Minneapolis, MN, USA). The extracted DNA was quantified with a DU 640 spectrophotometer (Beckman Instruments, Inc., Fullerton, CA, USA).

### Gene amplification and DHPLC

The coding region of the *APC *gene was divided into 28 segments, each of which was amplified separately using the polymerase chain reaction (PCR). The primer pairs used in this study were designed for both PCR and denaturing high performance liquid chromatography (DHPLC; Transgenomic, Inc., Omaha, NE, USA) as previously described [15]. All samples were amplified in 50-μl of a reaction mixture containing 50 ng genomic DNA, 10 mM Tris-HCl, 50 mM KCl, 2.5 mM MgCl_2_, 0.2 mM of each dNTP, 200 mM of each primer, and 0.15 U AmpliTaq Gold DNA polymerase (Applied Biosystems; Roche Molecular Systems, Inc., Branchburg, NJ, USA). The thermal cycling profile was composed of an initial denaturation step at 95°C for 10 min, 35 cycles of 30 sec of denaturation at 95°C, 30 sec of annealing at 55°C, and 1 min of extension at 72°C, with a final 10-min extension step at 72°C. DNA samples amplified from the same regions of *APC *genes were subjected to DHPLC and compared. When observed peaks differed in sharpness or appeared with more than 0.08 sec of difference from the main peak, potentially different DNA sequences were suspected. Such samples were therefore subjected to sequencing.

### DNA sequencing

Sequencing reactions were performed with the BigDye Terminator v3.1 Cycle Sequencing Kit (Applied Biosystems, Foster City, CA, USA). Each sequencing reaction was amplified in a 10-μl reaction mixture containing 10–30 ng amplified DNA. The thermal cycling profile consisted of an initial denaturation step at 95°C for 10 sec, 25 cycles of 10 sec of denaturation at 95°C, 5 sec of annealing at 50°C, and 1 min of extension at 60°C. Primers used for direct sequencing reactions were identical to those used in the amplification reactions. Nucleotide sequences of both strands were determined with an ABI Prism 3100 Genetic Analyzer (Applied Biosystems, Foster City, CA, USA) equipped with a long-read sequencing capillary and a POP-4 sequencing polymer.

### Data analysis

The sequence data collected from the ABI 3100 Genetic Analyzer were analyzed by software programs from Applied Biosystems. The analyzed sequence data were aligned through the use of BioEdit 7.0.5 software [16]. The Chi-square tests for Hardy-Weinberg equilibrium, the Chi-square and Fisher's exact tests for differences between the patient and health examination groups, and logistic regression analyses for odds ratios (without adjusting for other variables) were processed by SAS 9.1.

## Results

When the coding regions of *APC *genes of all 154 subjects enrolled in the study were sequenced, 12 SNPs and 1 deletion mutation were identified (Table 1). Of these variants, 8 SNPs were located within exon 15, 2 SNPs within exon 11, 1 SNP within exon 9, 1 SNP within exon 13, and 1 deletion within exon 10. Of the 12 SNPs, 9 are silent substitutions and 3 are responsible for the amino acid changes p.V1125A, p.S1126R and p.D1822V. Ten SNPs were identified in the health examination group and 2 of these SNPs, g.1488A>T and g.3378C>G, were not found in the patient group. Ten SNPs and 1 deletion mutation were found in the patient group and 3 of these variants, g.1050T>G, g.1378delG and g.4725A>G, were not found in the health examination group (Table 1).

The single base pair deletion, g.1378delG, which is located in exon 10, was found only in one individual in the patient group. This subject presented with a heterozygous genotype, confirmed by repeated forward and reverse sequencing. Prevalence of this mutation, which is reported here for the first time, was not significantly different between the patient and health examination groups.

Of the 3 observed missense substitutions, p.V1125A and p.S1126R represent variants not previously reported. p.V1125A was identified in three subjects in the patient group and in one subject in the health examination group, but p.S1126R was found only in the health examination group. However, the prevalence of these variants did not differ significantly between the patient and health examination groups. The characteristics of the subjects in the patient group with g.1378delG deletion or g.3374T>C (p.V1125A) are listed in Table 2. Prevalence of the p.D1822V variant, which has been observed previously [17-19], was significantly different between the patient and health examination groups. When this variant was analyzed further by determination of the odds ratio, a low risk for CRC was observed for subjects displaying the AT genotype (Table 3).

Of the 12 SNPs identified, only g.1458T>C and observed within the patient group displayed significant deviation from Hardy-Weinberg equilibrium (*p *= 0.002). To ascertain whether differences in the genotype distribution existed between the health examination group and patient group at these SNP sites, the Chi-square test and the Fisher's Exact test were performed. Significant differences were observed at g.1458T>C (*p *= 0.0012), g.4479G>A, (*p *= 0.0057), g.5268T>G (*p *= 0.0023). Analysis of the odds ratio for each genotype at these three sites revealed that g.1458T>C and g.4479G>A were significant and g.5268T>G was close to significant associated with a higher risk genotype (Table 3).

## Discussion

In this case-control study involving the screening of 74 healthy Taiwanese adults and 80 Taiwanese CRC patients for germline variants throughout the entire coding region of the *APC *gene, three new mutations were identified. No mutations reported in previous studies, including those at codons 1061 and 1309 which are considered the most common mutation sites, were identified. The absence in these Taiwanese subjects of the Caucasian-specific mutations p.E1317Q and p.I1307K that are known to cause missense changes is in accord with the findings of Guo *et al*. [20]. Additionally, Asian-specific variants such as the odd mutations reported in a Japanese study [21] were not displayed by any of the Taiwanese subjects in the present study. These observations are consistent with the proposals that the different presentations of the *APC *gene in these Taiwanese subjects are due to population differences and that the new mutations found in this study represent Taiwanese-specific hotspot mutations.

The three new mutations identified in the present study include a one base pair deletion and two missense substitutions. The one base pair deletion occurs at codon 460 of exon 10 and will cause a frameshift resulting in expression of a truncated protein. The two missense substitutions, p.V1125A and p.S1126R, occur in close proximity to the first 15-amino acid β-catenin binding repeat (amino acids 1136–1151) located at the center of the β-catenin down-regulation domain. Such substitutions may result in disruption of the putative cell signaling function of the APC protein; however, this needs to be demonstrated experimentally. It should be noted that all three mutations presented with low frequency in subject population of the present study and that the p.S1126R mutation was observed only in the health examination group. The sites at which these mutations occur, therefore, may not represent hotspots commonly associated with CRC. Nevertheless, the presence of these mutations may result in development of CRC. Long-term monitoring of persons who present with these mutations is therefore considered essential.

The frequency distribution of the 13 variants identified in the present study differed significantly between the health examination group and patient group at g.1458T>C, g.4479G>A, g.5268T>G and g.5465A>T (Table 1, p < 0.05 by the Chi-square test or Fisher's Exact test). Further analysis by logistic regression indicated that genotypes at g.1458T>C, g.4479G>A and g.5268T>G had high odds ratios for CRC (Table 3). These genotypes may therefore serve as markers predicting the development of CRC. The polymorphism p.D1822V, which was observed in the present study, is located in close proximity to the fifth APC cysteine-rich region β-catenin binding repeat (amino acids 1840–1866). p.D1822V is a missense variant previously shown to be a common polymorphism unrelated to the risk of developing CRC or colorectal adenoma [18,19]. However, two previous studies of CRC demonstrated significant gene-environment interactions between the p.D1822V polymorphism and consumption of a low-fat diet and postmenopausal hormone use [19,22]. Although no TT homozygotes for this site were identified in the present study, AT heterozygotes for this site were identified who had a low odds ratio for CRC (Table 3). The presence of a T allele at this site may therefore serve to protect against development of CRC.

Certain SNPs of *APC *genes were previously reported to be differentially distributed among different populations. Of the 10 SNPs identified in the present study the only SNP with a significantly different allele distribution, as determined by comparison with the East Asian population through use of the NCBI SNP databank (*p *<0.05, Chi-square test), was g.4479G>A. Further analysis revealed that an AA genotype for this SNP is associated with a higher risk for CRC. This observation implicates this genotype as a likely marker for CRC in the Taiwanese population. The other two SNPs, g.1458T>C and g.5268T>G, with higher risk genotypes for CRC were not found to have an allelic distribution different from other East Asian populations as determined through use of the NCBI SNP databank. These SNPs may therefore serve as markers for CRC specific to East Asian populations.

Investigations of the relationships existing between SNPs or germline mutations in the *APC *gene and occurrence of CRC should enable more accurate predictions of CRC. Furthermore, extensive screening of FAP patients should lead to a better understanding of the importance of events underlying development of CRC. Future studies of various high-risk *APC *gene mutations may also lead to improvements in therapy and, therefore, to increased survival of CRC patients.

## Conclusion

Among the *APC *genes of the 154 Taiwanese subjects examined in this case-control study three new mutations, but no previously reported mutations, were identified. Taiwanese subjects therefore appear to differ from other subjects with respect to the genotypic presentation of *APC*. Three high risk genotypes at 3 SNPs and one low risk genotype at codon 1822 were identified in this study. The latter was reported previously to have no association with CRC.

## Abbreviations

CRC, colorectal cancer; PCR, polymerase chain reaction; SNP, single nucleotide polymorphism; DHPLC, denaturing high performance liquid chromatography

## Competing interests

The author(s) declare that they have no competing interests.

## Authors' contributions

SPC carried out the acquisition of data, data analysis, drafted and wrote the manuscript. STT, YLH, YLC, YCC and CCW participated in the acquisition of data, sequence and wrote the manuscript. SZL, HJH, SWJ conceived of the study, and participated in its design and coordination and helped to draft the manuscript. All authors read and approved the final manuscript.

## Pre-publication history

The pre-publication history for this paper can be accessed here:



## Figures and Tables

**Table 1:** Genotype distributions for the health examination and patient groups.

Location	DNA and amino acid change	Validation	Health examination group allele distribution	Patient group allele distribution	p*
exon 9	g.1050T>G	SNP	TT = 74 GG = 0	TT = 79 GG = 0	NS^#^
	silent	unreported	TG = 0 HW^$ ^= 1	TG = 1 HW = 0.955	
exon 10	g.1378delG	deletion	GG = 74 -- = 0	GG = 79 -- = 0	NS
	p.E460fs	unreported	-G- = 0 HW = 1	-G- = 1 HW = 0.955	
exon 11	g.1458T>C	SNP	TT = 2 CC = 41	TT = 5 CC = 62	0.0012
	silent	rs2229992^§^	CT = 31 HW = 0.169	CT = 13 HW = 0.002	
exon 11	g.1488A>T	SNP	AA = 73 TT = 0	AA = 80 TT = 0	NS
	silent	rs9282599^§^	AT = 1 HW = 0.953	AT = 0 HW = 1	
exon 13	g.1635G>A	SNP	GG = 0 AA = 55	GG = 0 AA = 57	NS**
	silent	rs351771^§^	AG = 19 HW = 0.205	AG = 23 HW = 0.133	
exon 15	g.3374T>C	mutation	TT = 73 CC = 0	TT = 77 CC = 0	NS
	p.V1125A	unreported	TC = 1 HW = 0.953	CT = 3 HW = 0.864	
exon 15	g.3378C>G	mutation	CC = 73 GG = 0	CC = 80 GG = 0	NS
	p.S1126R	unreported	CG = 1 HW = 0.953	CG = 0 HW = 1	
exon 15	g.4479G>A	SNP	GG = 2 AA = 49	GG = 0 AA = 69	0.0057
	silent	rs41115^§^	AG = 23 HW = 0.718	AG = 11 HW = 0.509	
exon 15	g.4725A>G	SNP	AA = 74 GG = 0	AA = 79 GG = 0	NS
	silent	unreported	AG = 0 HW = 1	AG = 1 HW = 0.955	
exon 15	g.5034G>A	SNP	GG = 7 AA = 44	GG = 2 AA = 59	NS
	silent	rs42427^§^	AG = 23 HW = 0.141	AG = 19 HW = 0.753	
exon 15	g.5268T>G	SNP	TT = 7 GG = 44	TT = 0 GG = 63	0.0023
	silent	rs866006^§^	GT = 23 HW = 0.141	GT = 17 HW = 0.288	
exon 15	g.5465A>T	SNP	AA = 64 TT = 0	AA = 78 TT = 0	0.0145
	p.D1822V	rs459552^§^	AT = 10 HW = 0.533	AT = 2 HW = 0.910	
exon 15	g.5880G>A	SNP	GG = 1 AA = 51	GG = 0 AA = 57	NS
	silent	rs465899^§^	AG = 22 HW = 0.418	AG = 23 HW = 0.133	

§:The refSNP IDs from NCBI RefSNP Clusters are listed under reported SNPs.

$HW: *p *value of Hardy-Weinberg equilibrium obtained by Chi-square test.

#NS: Not significant.

*: The difference distribution of genotypes were examined by Chi-square test or Fisher's exact test.

**: The *p *value was obtained by Chi-square test, and others by Fisher's exact test.

**Table 2:** The characteristics of the patients with 1378delG or 3374T>C.

Patient No.	Mutation	Sex	Age	Tumor grade^§^	Tumor location
p11	g.1378delG	male	39	B	distal
p40	g.3374T>C	male	61	B	rectal
p27	g.3374T>C	male	52	B	proximal
p61	g.3374T>C	male	51	C	rectal

§: Duke's stage.

**Table 3:** The ORs for CRC of APC genotypes at g.1458T>C, g.4479G>A, g.5268T>G and g.5465A>T.

Variant	Genotype	Numbers in healthy/patient group	OR (95% CI^$)^	p*
g.1458T>C	TT	2/5	1.65 (0.31 – 8.93)	NS^#^
	TC	31/13	0.28 (0.13 – 0.59)	0.001
	CC	41/62	1	

g.4479G>A	GG	2/0	0	NS
	GA	23/11	0.34 (0.15 – 0.76)	0.009
	AA	49/69	1	

g.5268T>G	TT	7/0	0	NS
	TG	23/17	0.52 (0.25 – 1.08)	0.078**
	GG	44/63	1	

g.5465A>T	AA	64/78	1	
	AT	10/2	0.16 (0.03 – 0.78)	0.023
	TT	0/0		

$CI: Confidence interval.

*: The *p *value was obtained by logistic regression.

**: Close to significant.

#NS: Not significant.
